# Understanding barriers to the introduction of precision medicines in non-small cell lung cancer: A qualitative interview protocol

**DOI:** 10.12688/wellcomeopenres.13976.1

**Published:** 2018-03-08

**Authors:** Stuart Wright, Gavin Daker-White, William Newman, Katherine Payne

**Affiliations:** 1 Manchester Centre for Health Economics, Division of Population Health, Health Services Research & Primary Care, University of Manchester, Manchester, M13 9PL, UK; 2 Centre for Primary Care, Division of Population Health, Health Services Research & Primary Care, University of Manchester, Manchester, M13 9PL, UK; 3 Manchester Centre for Genomic Medicine, University of Manchester, Manchester, M13 9PL, UK

**Keywords:** Precision Medicine, stratified medicine, qualitative interviews, capacity, barriers, non-small cell lung cancer, cost effectiveness

## Abstract

**Background:** While precision medicines targeting genetic mutations and alterations in non-small cell lung cancer (NSCLC) have been available since 2010, their adoption into clinical practice has been slow. Evidence suggests that a number of barriers, such as insufficient clinician knowledge, a need for training of test providers, or a lack of specific clinical guidelines, may slow the implementation of precision in general. However, little attention has been given to the barriers to providing precision medicines in NSCLC. The purpose of this protocol is to outline the design for a qualitative interview study to identify the barriers and facilitators to the provision of precision medicines for NSCLC.

**Methods: **This study will use semi-structured interviews with clinicians (n=10), test providers (n=10), and service commissioners (n=10) to identify the perceived barriers and facilitators to providing historical, current, and future precision medicines in NSCLC. Participants will be identified through mailing list advertisements and snowball sampling. Recruitment will continue until data saturation, indicated by no new themes arising from the data. Interviews will be conducted by telephone to facilitate geographical diversity. The qualitative data will be analysed using a framework analysis with themes anticipated to relate to; relevant barriers to providing precision medicines, the impact of different barriers on medicine provision, changes in the ability to provide precision medicines over time, and strategies to facilitate the provision of precision medicines.

**Ethics: **This study has been approved by the University of Manchester Proportionate Review Research Ethics Committee (Reference number: 2017-1885-3619). Written consent will be obtained from all participants.

**Conclusion: **This study is the first to explore the barriers and facilitators to providing precision medicines for NSCLC in the English NHS. The findings will inform strategies to improve the implementation of future precision medicines. These findings will be disseminated in peer-reviewed publications and national and international conferences.

## Introduction

The concept of precision medicine is gaining increased attention as a potentially effective and cost-effective approach to the treatment of patients ( Hatz *et al.*, 2014; Payne *et al.*, 2018; Phillips *et al.*, 2014). Currently, the applied examples of precision medicine use a companion test-treat strategy to separate patients into groups according to the likelihood of responding to treatment or experiencing side effects. Medicines that use a companion test are now available for the management of non-small cell lung cancer (NSCLC) in clinical practice. The first example, gefitinib, was licensed in Europe in 2009, making it available for use in clinical practice. The medicine was then required to be made available to all eligible patients in the English NHS from 2010 with the approval of the drug as part of the National Institute for Health and Care Excellence (NICE) technology appraisal process ( National Institute for Health and Care Excellence, 2010). Gefitnib was appraised by NICE to provide sufficient benefits given its costs for patients with advanced NSCLC who had cancer which tested positive for mutations which lead to overexpression of epidermal growth factor receptor (EGFR). Further treatments for *EGFR* positive tumours were approved for recommendation by NICE in 2012 (erlotinib) and 2014 (afatanib) ( National Institute for Health and Care Excellence, 2014; National Institute for Health and Care Excellence, 2012). In 2012, crizotinib was licensed and subsequently was approved by NICE in 2014. This intervention involves targeting treatment using a test to detect a special type of mutation affecting the anaplastic lymphoma kinase ( *ALK*) gene called a translocation whereby chromosomes break and re-join creating fusion genes with increased activity. In 2016 ceritinib, that is also targeted to *ALK* mutations, was also approved by NICE ( National Institute for Health and Care Excellence, 2013; National Institute for Health and Care Excellence, 2016a). Treatments targeting the overexpression of programmed death ligand 1 (PD-L1) have been licenced and approved and treatments targeting *BRAF* gene mutations have a product license and are currently undergoing appraisal by NICE ( National Institute for Health and Care Excellence, 2016b; National Institute for Health and Care Excellence, 2016c).

When treatments are recommended for use in the NHS by NICE, service commissioners are legally required to provide patients with access to these medicines within three months of the positive recommendation ( NHS, 2015). Although the provision of companion diagnostics alongside precision medicines in cancer has recently become mandatory, historically this was not true ( National Institute for Health and Clinical Excellence, 2011; The British in Vitro Diagnostics Association *et al.*, 2016). Evidence produced by the charity Cancer Research UK has suggested that there is a significant lag in the provision of mutation testing from when the precision medicine is first licensed to all patients gaining ready access to the medicine in the NHS ( Cancer Research UK, 2015). The study, using a survey of 56 laboratories which were known to conduct molecular testing, estimated that in 2014, 48% of patients were not receiving mutation tests and this could mean that approximately 1,428 out of 3,007 patients who could have benefitted from *EGFR* targeting therapies were missing out. Prompt receipt of test results is required to inform clinical decision making but in some areas where testing was required to be made available for all patients, fewer than 50% of patients had *EGFR* test results available at their first consultation ( Evans *et al.*, 2013). Potential benefits from increasing the proportion of patients who receive a precision medicine have been improved life expectancy and fewer severe side effects than those on standard chemotherapies ( Banz *et al.*, 2011).

The availability of cancer somatic (tumour) mutation testing in the NHS has been repeatedly discussed during the appraisals of medicines using a test to direct treatment as part of the NICE appraisal process. In 2010, it was highlighted in the NICE appraisal of gefitinib that *EGFR* testing was not routinely conducted in the NHS, but it was said that the capacity to provide testing was present ( National Institute for Health and Care Excellence, 2010). In 2012, the assessment report for erlotinib stated that testing had become standard practice ( National Institute for Health and Care Excellence, 2012). However, the 2014 technology appraisal report for afatinib highlighted that there was still regional variation in the turnaround time for tests ( National Institute for Health and Care Excellence, 2014). As such, there is evidence that not all patients had access to precision medicines for NSCLC, or their requisite tests over four years after approval, despite the requirement of access being provided after 3 months. As a result, patients were not receiving interventions which could have provided improved length and quality of life which should have been available to them.

## Previous research

A study using face-to-face semi-structured interviews which explores how oncologists’ perceptions and work environment affect their use of genomic-targeted medicines is currently being undertaken in the United States ( Chen *et al.*, 2015). The published protocol for this study presents the results of a pilot study but it is difficult, however, to generalise outside the US setting that has a specific privately funded healthcare system. Under the remit of NHS England, which is funded by a tax-based healthcare system, all medicines recommended by NICE are legally required to be made available to patients ( NHS, 2015). Furthermore, in the United States, there is no obligation to provide treatments and decision making will likely be more devolved to clinicians and patients rather than the more guideline focussed UK.

There may be some commonalities in clinician experiences between the US and UK. Approximately a third of the ten oncology fellows interviewed in the pilot study were uncertain about guidelines regarding the use of precision treatments as second or third line treatments for lung cancer while a third of those interviewed were also uncertain regarding how to order testing. Common barriers to performing tests included insufficient tissue samples, the inconvenience of testing and the cost of testing. Facilitators of tests were the ease of testing and deciphering results, as well as patients having health insurance. The cost of treatment was mentioned as a barrier by a smaller number of clinicians. These findings highlight how differences in financing arrangements may impact on the use of precision medicine in oncology. For beneficial targeted treatments to be prescribed, test results need to be available in a timely manner. Delays in receiving test results were identified as a barrier to patients starting targeted therapies by half of the participants. These results mirror the findings of a US survey which found that the greatest perceived barrier to the use of precision medicines in practice was the cost of testing and targeted therapies ( Petersen *et al.*, 2014).

Other studies have sought to identify the barriers to precision medicine more generally. Taking account of this collective evidence base means the definition of precision medicine must also include tests for genetic predisposition for disease and tests for susceptibility to adverse events. In 2008, Newman and Payne identified that few clinical laboratories were offering pharmacogenetic testing services ( Newman & Payne, 2008). The timing of tests and coordination of testing with treatment was identified in qualitative interviews with stakeholders in breast cancer care as a key constraint of access to precision medicine alongside delays in testing whilst payer authorisation was sought ( Weldon *et al.*, 2012). In a 2013 study based in Canada, which used focus groups with physicians, the identified key relevant concerns about introducing precision medicine included: insufficient knowledge; a need for training of physicians; lack of specific guidelines and protocols for using tests; unequal access to testing due to socioeconomic differences; the financial burden of testing on public funds; additional time pressures that precision medicine will put on clinical practice; need for geneticist support after testing ( Najafzadeh *et al.*, 2013). The same authors also derived quantitative weights for the importance of different barriers in a subsequent study using a discrete choice experiment ( Najafzadeh *et al.*, 2012). The key attributes driving physicians’ preferences for using precision medicine were the availability of training and guidelines. Interestingly, this preference study also found two sub-groups with different types of preferences: one much more sensitive to the cost of the test than the other ( Najafzadeh *et al.*, 2012). Physicians in this group were more likely to be female.

In a systematic review of previous literature, with a particular focus on strategic reports from the European Commission funded PerMed – FP7 project, Horgan *et al.*, (2014) identified a wide range of constraints to introducing precision medicine in Europe ( Horgan *et al.*, 2014). Barriers included limited resources, test turnaround time, lack of health professional knowledge and communication. Furthermore, the authors also identified barriers relevant to patients including a lack of awareness and understanding of precision medicine and poor health literacy. Another significant barrier to implementing precision medicines across Europe will be understanding how reimbursement decisions can be made about such interventions given their unique properties ( Payne & Annemans, 2013).

Despite the number of studies investigating barriers to the uptake of precision medicine in general, there has been a paucity of research focussing on the delayed implementation of interventions targeting NSCLC. Furthermore, there have been no studies examining the barriers to implementing precision medicine in the context of treating NSCLC in the UK NHS. As the number of precision medicines approved for use in NSCLC continues to expand, it becomes increasingly urgent to understand how best to implement such interventions in order to ensure that all relevant patients have access to potentially life extending and improving treatments.

## Aim

The primary aim of this study is to explore the type and extent of barriers experienced by service providers and service commissioners when introducing licensed precision medicines for the treatment of NSCLC in relevant patient populations and individuals. Furthermore, a secondary aim is to identify strategies which have facilitated the improved provision of precision medicines for NSCLC in the English NHS.

## Objectives

This study has four objectives:

• To identify the types of perceived clinical and organisational barriers to providing licensed test-treat medicines indicated for the treatment of NSCLC to patients;
• To explore the potential impact of the identified different barriers to the provision of licensed test-treat medicines indicated for the treatment of NSCLC;
• To explore how the availability of existing licensed test-treat medicines indicated for the treatment of NSCLC has changed over time;
• To identify strategies which have been used to improve the availability of licensed test-treat medicines indicated for the treatment of NSCLC.

## Methods

This study will use semi-structured telephone interviews with clinicians, test providers, and service commissioners to identify the barriers to implementing licensed test-treat medicines indicated for the treatment of NSCLC. Previous research has shown that there have been issues with implementing precision medicine for lung cancer into the NHS but few have explored why this was the case for these medicines in particular ( Chen *et al.*, 2015). While quantitative analyses can assist in showing the number of patients not prescribed precision medicines it is useful to use qualitative methods to explore the reasons for this observation. Qualitative methods, such as semi-structured interviews, can be used to explore the thoughts, attitudes and opinions of those who were involved in implementing licensed test-treat medicines indicated for the treatment of NSCLC in clinical practice. In this study, semi-structured interviews will be used to understand the barriers to introducing licensed test-treat medicines indicated for the treatment of NSCLC. This approach also has the advantage of allowing the investigation of the perceived barriers of precision medicines introduced at different points in time by identifying the experiences and opinions of key stakeholders. This will allow the exploration of the potential for the English NHS to learn from previous implementation issues to improve future treatment provision.

### Sampling

The sampling frame will aim to identify stakeholders with experience of providing and introducing licensed test-treat medicines indicated for the treatment of NSCLC. While demand side factors, such as uptake of treatment or adherence to medicines, linked to patients’ preferences for treatment may also impede the implementation of precision medicine, the focus of these interviews is to identify the supply side capacity constraints. Therefore, patients will not be interviewed in this study. The relevant stakeholders will be drawn from two groups: service providers, for example clinicians, pathologists, and geneticists; and service commissioners which may include individuals who are members of care commissioning groups or involved in commissioning at the national level through NHS England. The principle service providers of interest are oncologists and respiratory physicians specialising in lung cancer, but also geneticists and pathologists who are key in providing examples of tests used in licensed test-treat medicines indicated for the treatment of NSCLC, such as *EGFR* and *ALK* testing and the emerging PD-L1 test.

Purposive sampling will be used to gain a diverse sample in terms of the setting and geographical location of testing and treatment ( Palinkas *et al.*, 2015). This characteristic is likely to be important in the context of introducing precision medicines as experiences may vary depending on the size and nature of hospitals and trusts. For example, mutation testing services may be more readily available in larger teaching hospitals with established links to laboratories. For smaller, rural hospitals there may be a greater logistical challenge in sending samples for testing and receiving results in a timely manner.

Service providers with over 7 years of NHS experience will be targeted as such individuals are likely to have direct experience of the introduction of *EGFR* and *ALK* testing and treatment as they were working in clinical practice.

The service commissioner sample will comprise hospital, regional and national level individuals involved with service commissioning and funding decisions. Examples of service commissioners may involve members of care commissioning groups, hospital finance staff and decision makers involved with national organisations, such as the National Institute for Health and Care Excellence (NICE). As in recruitment for the clinician sample, geographical diversity will be sought through purposive sampling and service commissioners will be required to have been in a relevant position when *EGFR* and *ALK* mutation based testing and treatment were introduced.

### Sample size

There are no defined rules for calculating sample size in qualitative studies ( Patton, 2002). In quantitative studies, a sufficient sample size is required to identify statistically significant differences in the variables of interest. However, qualitative interviews are aimed at identifying the breadth of experiences, thoughts, or opinions on a given subject. This study will therefore start with an approximate sample of 10 clinicians or test providers, and 10 service commissioners but sampling will continue iteratively until no new themes are arising from the collected data, otherwise known as inductive thematic saturation ( Saunders *et al.*, 2017).

### Recruitment

The clinician and test provider samples (n=20) will be recruited via the British Thoracic Oncology Group (BTOG) ( British Thoracic Oncology Group, 2017) and the Royal College of Pathologists (RCPath) ( Royal College of Pathologists, 2017). Details about the study and an invitation to participate will be circulated via the BTOG mailing list which currently has 2083 members and the RCPath that has over 11,000 members. Information regarding the study will be sent to participants using mailing lists, with contact details of the principal investigator provided for those interested in taking part. These individuals will then be sent more detailed information about the study.

Service commissioners (n=10) will be recruited using existing links and collaborations within the research team to identify an initial sample. Service commissioners will be directly sent an email including information about the study and the contact details of the principal investigator. Snowball sampling will be used for both samples whereby participants will be asked if they know any other individuals who meet the inclusion criteria who may be interested in taking part in the study ( Lewis-Beck *et al.*, 2004).

### Telephone interviews

A bespoke telephone interview schedule has been created to address the key research questions while remaining open enough to allow relevant new lines of enquiry to be explored. Due to the focus of this work on capturing a geographically diverse sample to represent heterogeneity in health care provision, telephone interviews will be used to collect qualitative data ( Musselwhite *et al.*, 2007; Novick, 2008). Semi-structured interview schedules will be created for each sample, informed by a review of previous economic evaluations of precision medicines (including health technology assessments) and consultation with two expert clinical advisors who are lung oncologists and a patient representative group (Roy Castle Lung Cancer Foundation). An initial draft interview schedule for the clinician sample is presented in Supplementary File 1. While the core questions for each interview schedule will be similar, there will be slight variations in the way questions are asked depending on the particular role of the interviewee. For example, clinicians will be asked primarily about their experience offering treatments to patients while for geneticists and pathologists the focus will be on offering testing. Interviews are expected to last approximately 1 hour.

All interviews will be audio-recorded and transcribed verbatim by an approved, contracted transcription company ( Associated Verbatim Reporters). Recordings will be sent via an encrypted data transfer.

### Data analysis

The aim of the data analysis is to identify the range of barriers which may prevent patients’ access to precision treatments for NSCLC, to determine which are the most important barriers, and to identify strategies to improve the implementation of precision treatments. The qualitative data will be analysed using a framework analysis facilitated by using the NVivo software ( QSR International, 2017)

Framework analysis is a five stage process involving; familiarisation, identifying a thematic framework, indexing, charting, mapping and interpretation ( Ritchie & Spencer, 1994). In the initial familiarisation stage, the researcher reads an initial set of the interviews in order to gain an understanding of the initial themes emerging from the data.

The initial key themes identified during the data familiarisation stage, alongside evidence from previous research, form an initial thematic framework against which the selection of data is sorted and collected ( Gale *et al.*, 2013). As semi-structured interviews are being used for this study, it is anticipated that many of the themes will originate in the questions contained in the interview schedule. As new themes emerge from the data, they are added to the framework. Each transcript is then indexed against these themes, with sections from the text which support different themes annotated for later retrieval. Charting brings the separate transcripts together to create a picture of the research as a whole ( Ritchie & Spencer, 1994). A chart is drawn up featuring the identified themes and potentially sub-headings for these themes. Information from each participant’s transcript which links to these themes is recorded in the chart, keeping the order of participants the same in each theme.

This analysis of qualitative evidence in a systematic way facilitates the discovery of patterns in the data while highlighting deviant cases for further investigation. In the context of this study, this will be the range of barriers which occur in providing and accessing test-treat medicines for NSCLC and views about which barriers were most significant in restricting the provision of precision medicines. However, the use of charting will also make clear the types of respondent referring to different topics which will serve to highlight the different perspectives of the availability of precision medicines.

### Data storage and anonymisation

Phone calls and recording will take place while the researcher is at The University of Manchester in an enclosed office. The recording device and memory card containing the interview recordings will be stored in a locked draw in a secure university office. The recordings will be saved onto an encrypted university computer, and the files password protected. Transcription of interviews will be conducted by a university approved company with secure file transfer protocols. Recorded interviews will be deleted from recording devices after they have been stored on a computer and anonymised and then destroyed completely at the end of the study. Interview transcripts will be stored for 10 years.

Anonymisation will be accomplished by removing references to participants’ names as well as any reference to information which could lead to identification of the participant such as the name of their place of work. When referencing data from the transcripts, generalised information regarding the participant will be provided to demonstrate their demographics whilst not allowing identification.

### Ethical concerns

This study has been approved by the University of Manchester Proportionate Review Research Ethics Committee (Reference number: 2017-1885-3619).

Clinicians and service commissioners who are interested in taking part in the study will be asked in the mailing list adverts to email or phone the research team to express an interest in taking part. The researchers will then email the potential participant a participant information sheet ( Supplementary File 2). After receiving an information sheet ( Supplementary File 2), potential participants will be given at least 24 hours to consider taking part in the study. If they agree to take part they will be asked to complete a written consent form and to return a copy to the researchers by post or email ( Supplementary File 3).

### Dissemination of findings

This study will form part of the PhD thesis for Stuart Wright. It is anticipated that this will be submitted in September 2019. All research participants will be emailed a summary of the main findings of this study after data analysis has been completed. The research team also plan to publish the full study in a peer-reviewed journal and present the results at relevant national and international conferences, for example the annual BTOG conference. In line with the ethical approval for this project, the raw qualitative data will not be made publically available.

### Remuneration

Participants will not receive remuneration for taking part in this interview study.

### Contribution of this study

The aims of this study are to identify barriers to providing precision medicines to patients and strategies which may be used to improve patient access to the medicines. As precision medicine is a rapidly expanding area and NICE continues to evaluate new examples of precision medicine in NSCLC, it is hoped that by learning from previous examples of slow implementation of such interventions, the rate at which new interventions are incorporated into the health service can be improved. This will ensure that patients have access to new treatments which offer the potential to improve their quality and quantity of life.

In addition to these broad aims, the results of this study will be directly used in the lead author’s PhD to inform economic models of example precision medicines in NSCLC. Currently economic evaluations conducted to determine the cost-effectiveness of such interventions do not take account of the barriers to their provision in the health service. It is feasible that if the costs and benefits of providing these interventions to patients are dependent on the level of implementation, then the cost-effectiveness of such treatments will also depend on their level of use in practice. It is therefore important to understand the barriers to using apparently cost-effective precision medicines to ensure that these are implemented in a manner which makes best use of the limited resources available in the health system. The lead author’s PhD will investigate how including these barriers impacts on cost-effectiveness estimates and the barriers identified in this study will be used in applied examples from NSCLC.

To date two interviews have been conducted for the study and recruitment is open for the clinician and service commissioner samples. Participants are being sought for pilot interviews in the test provider sample.

## Data availability

*All data underlying the results are available as part of the article and no additional source data are required.*

## Supplementary material

Supplementary File 1: Draft of the semi-structured telephone interview schedule

Supplementary File 2: Participant Information Sheet.

Supplementary File 3: Consent form for participants.
