# Supplementary material for: Understanding barriers to the introduction of precision medicines in non-small cell lung cancer: A qualitative interview protocol
Source: Wellcome Open Res. 2018 Mar 8;3:24. [Version 1] doi: 10.12688/wellcomeopenres.13976.1 (PMC5934686; doi:10.12688/wellcomeopenres.13976.1)
Supplement: Supplementary file 1 [file wellcomeopenres-3-15191-s0000.tgz › 728350af-6b7f-454a-8b52-f062b4ac0a90.docx]

**The semi-structured telephone interview schedule (draft)**

**Introduction**

**(Note: phrases in brackets and bold represent prompts for the interviewer)**

Thank you for agreeing to be interviewed.

I am a PhD student based in the Manchester Centre for Health Economics at The University of Manchester. I am funded by the Wellcome Trust.

I want to ask you some questions to explore your experience with using precision medicine to treat NSCLC.

The interview should take no longer than one hour and will be recorded. Firstly could you confirm that you have received and returned the consent form for this interview? Do you have any questions before we start?

**General**

How would you define precision medicine in the context of NSCLC?

Given this definition, could you list all of the currently licensed test-treat medicines for NSCLC which you are aware of?

**Probe: are you aware of any new examples of precision medicines currently being trialled**

Which of these have you used in clinical practice? (**link to questions below**)

**Experience of *EGFR* mutation testing and treatment**

1. Were you working in this or a similar role when *EGFR* targeting interventions were introduced into the NHS **(~2010)**?

2. Could you tell me about your memory of what happened when EGFR was first introduced and subsequent events?

Can you remember when you first prescribed an *EGFR* mutation targeting treatment?

- **(Were you aware of *EGFR* testing/treatments before they were approved? How did you hear of them?) (For example clinical trials, conferences etc)**
- **(How did you hear of them if you were not aware of these interventions before approval?)**
- **(Could you offer these interventions immediately after approval? What proportion of patients could receive these treatments?)**
- **(What factors prevented you from offering testing and treatment to all patients?) (Probe but do not lead with previously identified barriers from literature)**
- **(Which were the biggest constraints to offering and using *EGFR* based testing and treatments?)**

3. Do you currently prescribe treatments that require *EGFR* mutation testing?

4. Are you aware of any guidelines regarding *EGFR* mutation testing in the NHS? (**i.e. national, regional hospital guidelines)**

4b. Are you aware of any guidelines regarding *EGFR* mutation based treatment in the NHS? (**i.e. national, regional hospital guidelines)**

5. In your experience, do all patients who are currently eligible to receive *EGFR* mutation testing and treatment have access to these interventions?

- **(What factors prevent you from offering testing and treatment to all patients?) (probe but do not lead with previously identified barriers from literature)**
- **(Which are the biggest constraints to offering and using *EGFR* based testing and treatments?)**

6. Are you aware of any strategies to increase the number of patients receiving *EGFR* mutation targeting treatments?

7. Are there any strategies which you believe could be implemented to further increase the number of patients receiving *EGFR* targeting treatments in the future?

**Experience of *ALK* mutation testing and treatment**

1. Were you working in this or a similar role when *ALK* targeting interventions were introduced into the NHS **(~2010)**?

2. Could you tell me about your memory of what happened when *ALK* was first introduced and subsequent events?

Can you remember when you first prescribed an *ALK* mutation targeting treatment? (**see prompts for *EGFR***)

3. Do you currently prescribe treatments that require *ALK* mutation testing?

4. Are you aware of any guidelines regarding *ALK* mutation testing in the NHS? (**i.e. national, regional hospital guidelines)**

4b. Are you aware of any guidelines regarding *ALK* mutation based treatment in the NHS? (**i.e. national, regional hospital guidelines)**

5. In your experience, do all patients who are currently eligible to receive *ALK* mutation testing and treatment have access to these interventions? (**see prompts for *EGFR***)

6. Are you aware of any strategies to increase the number of patients receiving *EGFR* mutation targeting treatments?

7. Are there any strategies which you believe could be implemented to further increase the number of patients receiving *EGFR* targeting treatments in the future?

**Other key precision medicines**

**(Repeat above questions for other commonly used precision medicines identified by respondent)**

**Precision medicines in trials**

Do you believe there may be factors which will slow the implementation of these newer examples of precision medicines?

To what extent do you believe previous experience will facilitate the implementation of future precision medicines? (e.g. individual, regional, national strategies)

**Closing questions**

Are there any other comments you would like to make about any difficulties you have had in offering precision medicines for NSCLC?

Do you have any other questions about this study?

Thank you. I will now stop the recording

Can I now ask you some questions about yourself?

In which NHS hospital and trust do you work?

What is your job title? (**prompt for grade if not stated**)

How many years of experience do you have in this role?
