# Supplementary material for: Understanding barriers to the introduction of precision medicines in non-small cell lung cancer: A qualitative interview protocol
Source: Wellcome Open Res. 2018 Mar 8;3:24. [Version 1] doi: 10.12688/wellcomeopenres.13976.1 (PMC5934686; doi:10.12688/wellcomeopenres.13976.1)
Supplement: Supplementary file 2 [file wellcomeopenres-3-15191-s0001.tgz › 3ba5ebfe-e745-474c-ad9d-6fe351251337.docx]

**Understanding Barriers to the Introduction of Precision Medicines in Lung Cancer**

**Participant Information Sheet**

You are being invited to take part in a research study being conducted as part of a PhD project seeking to understand how economic evaluations of precision medicines can incorporate capacity constraints. Before you decide whether to take part, it is important for you to understand why the research is being done and what it will involve. Please take time to read the following information carefully and discuss it with others if you wish. Please ask if there is anything that is not clear or if you would like more information. Take time to decide whether or not you wish to take part. Thank you for taking the time to read this.

**Who will conduct the research?**

This research is being conducted by Mr Stuart Wright MSc, Manchester Centre for Health Economics, as part of his PhD programme of study.

**What is the purpose of the research?**

This study is focussing on the introduction of precision medicines for the treatment of non-small cell lung cancer (NSCLC). While such treatments have been approved for use in the National Health Service since 2010 (epidermal growth factor receptor tyrosine kinase inhibitors) and 2014 (anaplastic lymphoma kinase inhibitors), their use in clinical practice was slow to develop. This meant that many patients who may have benefitted from these treatments did not have access to them. The aim of this research is to identify the barriers that slowed the introduction of precision treatments for non-small cell lung cancer. Identifying these barriers may inform the implementation of new precision medicines in non-small cell lung cancer such as programmed death ligand 1 and B-Raf inhibitors.

**Why have I been chosen?**

You have been chosen as you are either a clinician or service commissioner involved in the provision or commissioning of precision medicines in non-small cell lung cancer.

**What would I be asked to do if I took part?**

A researcher (Stuart Wright), will interview you by telephone about your experience in providing precision medicines for non-small cell lung cancer. The interview will last approximately 1 hour and will be audio-recorded. However, your details will remain anonymous at all times.

In the interview we would like to hear about:

1. Your experience of the introduction of testing and treatments for patients with *EGFR* and/or *ALK* mutated non-small cell lung cancer.
2. The barriers that you experienced in providing these precision treatments
3. What strategies you used to overcome the barriers to using precision treatments
4. Whether any barriers still remain to using precision treatments for non-small cell lung cancer.

**What happens to the data collected?**

The data from the combined interview transcripts in this study will be used to address a number of objectives:

1. To identify the range of clinical and organisational barriers to patients receiving approved and recommended testing and treatments for NSCLC.
2. To explore the relative impact of different barriers on patient access to testing and treatments for NSCLC.
3. To explore how the availability of existing approved and recommended testing and treatments for NSCLC developed over time.
4. To identify strategies which have been used to improve the availability of testing and treatments for NSCLC over time.

This will be achieved by using a qualitative analysis technique known as framework analysis. Data is analysed as it is collected and early themes in the interviews are identified. These key themes, alongside evidence from previous research, form an initial thematic framework against which the selection of data is sorted and collected. As new themes emerge from the data, they are added to the framework.

In the context of this study, these themes will be the range of barriers which occur in providing and accessing precision medicines in NSCLC as well as views about the importance of each issue. It is anticipated that the results of this study will help to inform the introduction of future precision medicines by using learning from previous treatments to avoid future barriers.

**How is anonymity maintained?**

Identifiable data will be stored on university computers to allow the researcher to link participants responses to their demographic information. A private transcription company will be used to transcribe data from recordings to text. The recordings will be sent using encrypted transfers and no participant identifiable data will be provided to the company. Furthermore, in reporting and publishing the results of the study, no participant identifying information will be included in the document.

Study data and material may be looked at by individuals from the University of Manchester, from regulatory authorities or from the NHS Trust, for monitoring and auditing purposes and this may well include access to personal information.

Data will be archived according to the University of Manchester's policy which is currently 5 years. Consent forms will be retained as essential documents, but items such as contact details will be deleted as soon as they are no longer required.

**What happens if I do not want to take part or if I change my mind?**

It is up to you to decide whether or not to take part. If you do decide to take part you will be given this information sheet to keep and be asked to sign a consent form. If you decide to take part you are still free to withdraw up to the time of publication of the study in a peer reviewed journal or the student’s final thesis, without giving a reason and without detriment to yourself.

**Will I be paid for participating in the research?**

You will not be paid for taking part in this study.

**What is the duration of the research?**

This study will involve 1 telephone interview which will be approximately 1 hour in length

**Where will the research be conducted?**

Interviews will be conducted by telephone. The researcher will be located at the University of Manchester and will telephone from a private office.

**Will the outcomes of the research be published?**

The outcomes of this research will constitute a part of the student’s PhD thesis. It is anticipated that this will be submitted in September 2019. Furthermore, the researchers will seek to publish the results of this research in a peer-reviewed journal.

**Who has reviewed the research project?**

This research has been reviewed by the researchers supervisors (Professor Katherine Payne and Dr Gavin Daker-White) and an overview of the research has been reviewed by an independent member of the student’s centre (Mr Niall Davison).

This project has been reviewed by the University of Manchester Proportionate Research Ethics Committee.

**What if something goes wrong?**

You are free to withdraw from the study at any time. No reasons need to be given. Any taped or paper record of the interview and your contact details will be destroyed.

If you are worried about any part of this study please contact the research team. You can phone Stuart Wright on 0161 306 7970 or Professor Katherine Payne on 0161 306 7906.

**What if I want to make a complaint?**

**Minor complaints**

If you have a minor complaint then you need to contact the researcher(s) in the first instance. We will respond to any complaints about the study. **To complain you can telephone Professor Katherine Payne on 0161 306 7906 or email** [**Katherine.payne@manchester.ac.uk**](mailto:Katherine.payne@manchester.ac.uk)**.**

**Formal Complaints**

**If you wish to make a formal complaint or if you are not satisfied with the response you have gained from the researchers in the first instance then please contact** the Research Governance and Integrity Manager, Research Office, Christie Building, University of Manchester, Oxford Road, Manchester, M13 9PL, by emailing: [research.complaints@manchester.ac.uk](mailto:research.complaints@manchester.ac.uk)  or by telephoning 0161 275 2674 or 275 2046.

**What Do I Do Now?**

If you have any queries about the study or if you are interested in taking part then please contact the researchers:

**STUART WRIGHT**

**EMAIL:** [**stuart.wright-2@manchester.ac.uk**](mailto:stuart.wright-2@manchester.ac.uk)

**TELEPHONE: 0161 306 7970**

**This Project Has Been Approved by the University of Manchester’s Research Ethics Committee [Reference: 2017-1885-3619].**
