# Supplementary material for: Understanding barriers to the introduction of precision medicines in non-small cell lung cancer: A qualitative interview protocol
Source: Wellcome Open Res. 2018 Mar 8;3:24. [Version 1] doi: 10.12688/wellcomeopenres.13976.1 (PMC5934686; doi:10.12688/wellcomeopenres.13976.1)
Supplement: Supplementary file 3 [file wellcomeopenres-3-15191-s0002.tgz › 1eb73702-203a-46ca-afdc-77f035603168.docx]

**Staff Interview Consent Form**

**Title of project:** Understanding Barriers to the Introduction of Precision Medicines in Lung Cancer

Please initial box

| 1. I confirm that I have read the attached information sheet on the above project and have had the opportunity to consider the information and ask questions and had these answered satisfactorily. |  |
| --- | --- |
| 2. I understand that my participation in the study is voluntary and that I am free to withdraw:-  * at any time  * without having to give a reason for withdrawing  * and without detriment to myself |  |
| 3. I understand that the interview will be audio recorded. |  |
| 4. I agree to the use of anonymous quotations from this interview in research outputs arising from the study |  |
| 5. I understand that data collected during the study may be looked at by individuals from the University of Manchester, from regulatory authorities or from the NHS Trust, where it is relevant. I give permission for these individuals to have access to my records. |  |
| 6. I agree to take part in this study |  |

**Name of participant:** ……….…..……..…… **Signed:** ................................ **Date:** ..................

**Name of researcher:** ………...…………..… **Signed:** ................................ **Date:** ..................

**This project has been approved by the University of Manchester Research Ethics Committee**
