# Supplementary material for: Understanding barriers to the introduction of precision medicines in non-small cell lung cancer: A qualitative interview protocol
Source: Wellcome Open Res. 2018 Mar 8;3:24. [Version 1] doi: 10.12688/wellcomeopenres.13976.1 (PMC5934686; doi:10.12688/wellcomeopenres.13976.1)
Supplement: Peer review history

# Peer review: Referee response for version 1

**DOI:** 10.21956/wellcomeopenres.15191.r32852

**Published:** 2018-05-02

**Authors:** Tomris Cesuroglu

**Affiliations:** 1 Athena Institute for Research on Innovation and Communication in Health and Life Sciences, Vrije Universiteit Amsterdam, Amsterdam, Netherlands

Wright et al. present an interesting study protocol that aim to investigate the barriers experienced by service providers (clinicians and test providers) and service commissioners towards introduction of licensed and approved precision medicines for treatment of NSCLC. The study investigates the past and present experiences, as well as looking into potential future situations. The findings will provide important input to the planned upcoming studies on how including the identified barriers impacts the cost-effectiveness estimates (to be carried out in the context of the PhD of the lead author).

 The rationale for and objectives of the study are clearly described. The perspective taken is also clear: focus is on the supply side constraints, rather than patient experiences.

 The study design is appropriate for the aim of the study, as it involves interviewing with key stakeholders on the service provision and service commissioning sides. Nevertheless, there is a potential area of improvement, which is including a stakeholder analysis in this study, or adjacent to it.

 Barriers involved in implementation of services in health systems do not only involve technical constraints. They are usually rooted in how the health system, including services, is organized. Stakeholders play a crucial role in how current status of service provision is shaped, and if and how new services can be introduced.

 At this point, using stakeholder analysis to understand the power and interest of different stakeholders in introduction of precision medicines in treatment of NSCLC can provide valuable information. The stakeholder map including the power and interest grid can potentially provide the landscape and help to understand why the identified barriers exist. Based on this information, strategies can be developed to overcome the barriers, as this is also a part of the study aims.

 The stakeholder analysis can be done retrospectively, i.e. to understand the status in past cases of EGFR and ALK and how this influenced their introduction into services, and also to understand the current (and potential future) cases. Stakeholder analysis is ideally carried out in the initiation and phase. But at this moment, it can also be incorporated to the existing protocol with additional questions in the topic guide for identification of stakeholders and their potential power and interests.

 The authors may think that they are well aware of the stakeholder landscape in English NHS and are already considering these in the study. Nevertheless, it should be considered that a structured analysis on stakeholders has the potential to provide further data and insights, enhancing their analysis approach (framework analysis). A stakeholder analysis may also provide data partially on the issues raised by the other reviewer, Brett Doble, such as how the pathology labs, as stakeholders, are organized.

 The following article provides a good overview of stakeholder analysis method, involving mainly the power and interest of the stakeholders: Bryson, J. M. (2004). What to do when stakeholders matter: stakeholder identification and analysis techniques. Public management review, 6(1), 21-53. Doi:  10.1080/14719030410001675722

 More detailed guides, involving stakeholders’ knowledge, position, alliances and resources on the matter, in addition to power and interest, can also be used (e.g. the WHO's Stakeholder Analysis Guidelines).

 The study protocol provides a detailed description of methods. I agree with the other reviewer’s suggestions for elaboration of potential biases, as well as strategies to limit the effects of them.

I have read this submission. I believe that I have an appropriate level of expertise to confirm that it is of an acceptable scientific standard.

# Peer review: Referee response for version 1

**DOI:** 10.21956/wellcomeopenres.15191.r31712

**Published:** 2018-03-19

**Authors:** Brett Doble

**Affiliations:** 1 Health Economics Research Centre, Nuffield Department of Population Health, University of Oxford, Oxford, UK

Wright et al. propose a very interesting study that looks to understand past, present and future barriers to the clinical implementation of what they term ‘precision medicine’ in non-small cell lung cancer (NSCLC) within the English National Health Service (NHS). To achieve this, they will use a number of semi-structured qualitative interviews with clinicians, test providers and service commissioners who will have likely been previously exposed to the challenges in implementing such approaches to care.

 Overall, the protocol clearly articulates the main aims of the research and details appropriate methods to answer the research question. I do, however, suggest a few issues that the authors might wish to consider to improve the study as outlined below:

 The authors have chosen to specifically focus on implementation issues for ‘precision medicine’ in NSCLC and have been quite prescriptive in the justification for their study (i.e., ‘no such evidence exists for this type of cancer). While I agree that this type of cancer provides an excellent case study to investigate such issues I wonder to what extent implementation issues are cancer-type specific and not just largely applicable to ‘precision medicine’ in all cancer types more generally. For example, I imagine that similar implementation issues were experienced when HER2 testing and trastuzumab treatment became available and that this may have already been the focus of considerable research. I do recognise that existing research might not be within the English context, but there may still be lessons that can be learned from such research to further focus your questions within the proposed qualitative interviews.

 I suggest that the authors attempt to highlight why implementation issues in NSCLC are potentially different from other types of cancer. For example, is it because multiple test and targeted treatments are available at first-line? Or potentially the fact that most NSCLC patients present to the clinic with late stage disease compared to other cancer types, which may affect implementation issues for testing? It would be helpful if the authors indicate more clearly how their research has been designed to specifically target discussion of these unique issues rather than just obtain the same discussion on standard challenges that have been previously identified when implementing precision medicine in general.

 Further to my point above, after reviewing the interview schedule it seems the questions being asked of the participants are very focused on the potentially outdated concept of single mutation tests and associated targeted treatments. One of the most unique things about precision medicine in NSCLC is the fact that multiplex testing potentially offers value in a first-line setting. This is highlighted in the authors protocol as they state that multiple genomic alterations are available to potentially guide first-line treatment (e.g., erlotinib and crizotinib). Given limited tumour tissue from biopsies it might be necessary that *EGFR* and *ALK* testing occur at the same time using a single test. I recognise that the UK might not be at this stage yet, but I think it might be useful to introduce a question/prompt concerning multiplex testing if this information does not come out in the questions concerning implementation of newer precision medicines.

 I think the previous research section of the protocol could be improved to be more comprehensive of the entire evidence base. I would specifically look for evidence concerning implementation issues with HER2 testing and trastuzumab as I mentioned above as well as the fact that there has also been some research on implementation of genome sequencing in clinical practice that may be very closely related to the issues the authors are trying to identify. Furthermore, I would also suggest that the authors attempt to look at research conducted by sociologists in this area as I identified a number of relevant studies with just a quick search that contain relevant insights, which may be used to better focus questions during the proposed qualitative interviews:

 Samuel, G.N. and Farsides B. The UK’s 100,000 Genomes Project: manifesting policymakers’ expectations. New Genetics and Society. 2017

 Day, S., et al. Stratified, precision or personalised medicine? Cancer services in the real world of a London hospital. Sociology of Health & Illness. 2017; 39(1):143-158.

 In addition, I wonder if the authors have considered taking a broader approach to understanding barriers to implementation by conducting ethnographic research, in which the interviews would form just one aspect of the data collection. I know this might be beyond the scope of this initial study, but it might be something to considering moving forward in order to put the results of the interviews into better context. One of the biggest implementation issues in the English NHS is likely to be technical issues in how tumour samples are collected and stored. This issue is further compounded by the fact that testing technology as well as knowledge concerning the genomics of cancer can rapidly change and that there are a number of different tests available for tumour genomic testing in lung cancer. To ensure uptake of testing, the NHS will require a huge organisational, professional and culture shift as trying to change how tissue is handled is a core issue in how pathology departments work in the NHS (e.g., genomic tests in cancer require rapid turnaround time, which traditionally has not been required). Fundamentally, it comes down to whether or not pathology labs share the same vision with policy makers and are they likely to oppose changes to their current model of pathology testing. This of course is also compounded by the lack of resources within the NHS to accommodate transformational change. To get at these issues you could potentially conduct observations at different testing site with England. The pathway from obtaining a biopsy sample to the sample being prepared and processed through testing and final generation of the results could be directly observed and details of the process recorded. Such methods would enable a more complete interpretation of implementation issues as the interview material could be extended by observations of the testing pathway at different sites, while fieldwork notes could be elaborated in light of individual views and explanations obtained in the interviews. This is just a suggestion as to how you could further build on the research proposed in the protocol.

 Finally, I suggest that the authors add some discussion in the protocol with regards to potential bias that may affect their results. For example, it would be helpful if the authors could comment on how they might look to address self-selection bias in their participant recruitment strategy, given participants most interested in this topic will be most likely to agree to participate in the study, particularly for the clinician sample. Will the authors make any attempt to engage with stakeholders that are not so keen on implementing precision medicine in NSCLC? Furthermore, on review of the interview schedule I noticed that some of the questions are asking participants to recall events that happened close to 10 years ago. Recall bias is likely to be an issue here, do the authors have any strategies to limit the effect on this on results (e.g., potentially through the use of prompts)?

I have read this submission. I believe that I have an appropriate level of expertise to confirm that it is of an acceptable scientific standard.
